# Proteomic and Carbonylation Profile Analysis of Rat Skeletal Muscles following Acute Swimming Exercise

**DOI:** 10.1371/journal.pone.0071839

**Published:** 2013-08-13

**Authors:** Francesca Magherini, Tania Gamberi, Laura Pietrovito, Tania Fiaschi, Luca Bini, Fabio Esposito, Marina Marini, Provvidenza Maria Abruzzo, Massimo Gulisano, Alessandra Modesti

**Affiliations:** 1 Department of Experimental and Clinical Biomedical Sciences, University of Florence, Florence, Italy; 2 Department of Life Sciences, University of Siena, Siena, Italy; 3 Department of Biomedical Sciences for Health University of Milan, Milan, Italy; 4 Department of Experimental, Diagnostic, and Specialty Medicine, University of Bologna, Bologna, Italy; 5 Don Carlo Gnocchi Foundation ONLUS, Milan, Italy; 6 Department of Experimental and Clinical Medicine, University of Florence, Florence, Italy; University of Pecs Medical School, Hungary

## Abstract

Previous studies by us and other groups characterized protein expression variation following long-term moderate training, whereas the effects of single bursts of exercise are less known. Making use of a proteomic approach, we investigated the effects of acute swimming exercise (ASE) on protein expression and carbonylation patterns in two hind limb muscles: the Extensor Digitorum Longus (EDL) and the Soleus, mostly composed of fast-twitch and slow-twitch fibres, respectively. Carbonylation is one of the most common oxidative modifications of proteins and a marker of oxidative stress. In fact, several studies suggest that physical activity and the consequent increase in oxygen consumption can lead to increase in reactive oxygen and nitrogen species (RONS) production, hence the interest in examining the impact of RONS on skeletal muscle proteins following ASE. Results indicate that protein expression is unaffected by ASE in both muscle types. Unexpectedly, the protein carbonylation level was reduced following ASE. In particular, the analysis found 31 and 5 spots, in Soleus and EDL muscles respectively, whose carbonylation is reduced after ASE. Lipid peroxidation levels in Soleus were markedly reduced as well. Most of the decarbonylated proteins are involved either in the regulation of muscle contractions or in the regulation of energy metabolism. A number of hypotheses may be advanced to account for such results, which will be addressed in future studies.

## Introduction

Understanding how different types of exercise affect skeletal muscle is very important because of the implications for health; moreover, such knowledge may contribute to the improvement of training programs. Many different training protocols have been used in studies involving animal models, including treadmill exercise and swimming exercise. Duration and intensity of training can be modulated in order to obtain a short-term exercise (usually 3–5 consecutive days of exercise) or a long–term exercise (ranging from a few weeks to months). Furthermore, studies on the effects of strenuous exercise and single bouts of exercise have been performed. The link between different types of exercise and muscle modification has been studied at different but coordinated levels, ranging from global analysis with proteomic techniques [1] to more detailed approaches such as analysis of signal transduction pathways. Most studies point out the central role of reactive oxygen (ROS) and nitrogen species (RNS) in muscle plasticity and damage [2]–[4]. ROS are normally produced at low levels and are required for normal force production. Physical activity and the consequent increase in oxygen consumption can lead to a temporary unbalance between ROS production and disposal. Understanding of the benefits or damages that an appropriate or inappropriate exercise can induce takes advantage of understanding of mechanical damage as well as of the role of ROS in signal transduction and in molecular target oxidation. Previous work shows that acute exercise may cause oxidation of sarcoplasmic reticulum Calcium-handling systems [5] and suggests that oxidative stress associated with strenuous exercise may be involved in muscle contractile dysfunction and fatigue [6], [7]. Unbalanced ROS levels can induce oxidation of DNA, lipids and proteins. Protein side-chain functional groups are estimated to capture 50–75% of all highly reactive oxygen species [8]. ROS can induce reversible or irreversible oxidation of protein targets. Cysteine residues, for example, exist not only in fully reduced form (−SH) but also in different oxidized states that are partially reversible and can change relatively to the cell’s redox state. The cysteine residues have a double role: i) protection from irreversible damage; ii) modulation of protein function [9]–[12]. The introduction of carbonyl groups into proteins is another oxidative, non-enzymatic modification that occurs either by direct interaction with ROS or indirectly through the peroxidation of lipids that subsequently oxidize the proteins [13]. Protein carbonylation can result in the unfolding or alteration of protein structure and function [14]. The evaluation of carbonyl groups is thought to be a good estimate of the extent of oxidative damage associated with aging, disease, toxic processes and physical exercise [15]–[18]. The carbonylation process is considered a modification that cannot be reversed by the enzymatic repair machinery of the cell as it causes oxidised proteins to be sent to the proteasome for degradation [19]. However, Wong C.M. *et al.* [20], [21]. discovered that not all carbonylated proteins are degraded by the proteasome and suggested that protein carbonylation and decarbonylation may serve as a mechanism of signal transduction. Their preliminary studies contemplate the possibility that protein carbonylation/decarbonylation might play a role in the redox signalling mechanism. A significant increase in muscle protein carbonylation following prolonged inactivity [22], physical exercise [23]–[25], and during aging [26], [27] has been observed in different tissues including muscle, nervous system and plasma. In previous papers, we identified several proteins that undergo an increase in carbonylation levels after physical exercise in both rat skeletal muscles [23] and human plasma [28]. Despite these variations, we demonstrated that a basal level of protein carbonylation is present both in skeletal muscle and in plasma, in keeping with several other studies [28]–[31]. This probably means that this phenomenon transcends physical exercise and exogenous oxidative stress and that a basal level of carbonylation is present and well tolerated in mammalian cells. The aim of this investigation was to study skeletal muscle protein carbonylation patterns in rats following acute swimming exercise (ASE). Swimming exercise has been widely used to study biochemical changes associated with forced physical activity in rodents and may help to understand the mechanisms evoking changes in proteins carbonylation. We analysed two hind limb muscles which are characterized by different fibre types: the Extensor Digitorum Longus (EDL) muscle which is composed of fast-twitch fibres and the Soleus muscle which is composed of slow-twitch fibres [32]. The carbonylated protein pattern was analysed by two-dimensional gel electrophoresis (2D-GE) followed by Western Blot (WB) with anti-dinitrophenyl hydrazone (DNP) antibodies. Carbonylated proteins were identified by mass spectrometry. Several enzymes and muscle contractile proteins show a reduced carbonylation level, thus suggesting a possible activation of protein turnover and/or decarbonylation system subsequent to a single acute bout of exercise.

## Materials and Methods

### Animal Models and Exercise

In our experiment, we used male albino Sprague-Dowley rats, aged two months at entry. Rats were housed in individual cages and fed standard diet without limitations. Room temperature was kept at 21±2°C and 12 hours of light were automatically alternated with 12 hours of dark. The Milan University Committee for the Use of Laboratory Animals approved animal handling, training protocol and mode of sacrifice. Ten rats were divided in two groups: five for the sedentary control (C) group and five for the acute swimming exercise group (ASE). Rats in this last group were individually placed in a deep basin (40 cm deep, 50 cm diameter) filled with lukewarm water (37±2°C) and devoid of holding devices, so that they were compelled to swim. They were constantly watched and forced to resume swimming in case they managed to float remaining motionless. The duration of exercise was 30 min. It was previously shown that swimming is an endurance type of exercise and that 30 minutes of swimming are very exhausting for untrained rats [33]. Moreover, swimming is the only kind of exhausting exercise we were able to devise since untrained rats refuse to run on a treadmill; for this reason, we adopted it in previous studies [34], [35]. Sixty min after exercise cessation rats were deeply anesthetized (100 mg/kg ip heparinized sodium thiopental) and were sacrificed. The hind limbs were opened and Soleus and Extensor Digitorum Longus (EDL) muscles were isolated. The muscles were immediately frozen in liquid nitrogen and stored at −80°C until use.

### Muscle Processing and 2D-GE

Frozen muscles were ground in dry ice in a cooled mortar, suspended in lysis buffer (50 mM Tris-HCl pH 7.0, 150 mM NaCl, 2 mM EGTA, 100 mM NaF, 1% (v/v) NP-40, 0.5% (w/v) deoxycholate, 0.1% (w/v) SDS containing a cocktail of protease inhibitors (Sigma) and solubilised by sonication on ice. After centrifugation, proteins were precipitated following a chloroform/methanol protocol [36] and resuspended in 8 M urea, 4% (w/v) CHAPS, 65 mM DTT. Isoeletric focusing **(**IEF) was carried out on 18 cm IPG-strips pH 3–10 NL (GE Healthcare, Uppsala, Sweden) and achieved using an Ettan IPGphor™ system (GE Healthcare). Protein samples (60 µg and 750 µg for analytical and preparative gels respectively) were loaded at approximately 2 cm from the anode by cup loading in the Ettan IPGphor Cup Loading Manifold™ (GE Healthcare) after the rehydration of the IPG strips with 350 µl of rehydration solution (8 M urea, 2% (w/v) CHAPS, 0.5% (w/v) DTE) supplemented with 0.5% (v/v) carrier ampholyte and a trace of bromophenol blue. The strips were focused at 16°C according to the following electrical conditions: 200 V for 1 h, 300 V for 1 h, from 300 to 3,500 V in 30 min, 3,500 V for 4 h, 5,000 for 2 h, from 5,000 to 8,000 V in 30 min, and 8,000 V until a total of 80,000 V/h was reached, with a limiting current of 50 µA/strip. After focusing, analytical and preparative IPG strips were equilibrated for 10 min in 6 M urea, 30% (v/v) glycerol, 2% (w/v) SDS, 2% (w/v) DTT in 0.05 M Tris-HCl buffer, pH 6.8, and subsequently for 10 min in the same buffer solution where DTT was substituted with 2.5% (w/v) iodoacetamide. The equilibrated strips were placed on top of 9−16% polyacrylamide linear gradient gels (18 cm×20 cm×1.5 mm) and embedded in 0.5% heated low-melting agarose in SDS electrophoresis running buffer (25 mM Tris, 192 mM glycine, 0.1% (w/v) SDS, pH 8.3). The methylenebisacrilamide was the cross-linker used in the 9–16% gradient. SDS-PAGE was performed in a PROTEAN II xi cell gel electrophoresis unit (Bio-Rad) at 10°C and at 40 mA/gel constant current, until the dye front reached the bottom of the gel. Analytical gels were stained with ammoniacal silver nitrate as previously described [37]; MS-preparative gels were stained with colloidal Coomassie [38].

### Derivatization and Immunodetection of Protein Carbonyls (Oxyblot)

After first dimension electrophoresis, protein carbonyls were derivatized to DNP (2,4-dinitrophenylhydrazone) by incubating IPG strips in 10 mM DNPH (2,4-dinitrophenylhydrazine, Sigma-Aldrich) dissolved in 2 N HCl, for 20 min at room temperature [39]. Following derivatization, the marked IPG strips were washed with 6 M Urea, 20% (v/v) Glycerol, 1% (w/v) SDS, 150 mM Tris-HCl, pH 6.8, then they were reduced, alkylated and run on 9–16% polyacrylamide linear gradient gels (18 cm×20 cm×1.5 mm). After running, gels were blotted overnight on Polyvinylidene Fluoride (PVDF) membrane. The PVDF membranes were incubated for three hours at 4°C with the primary antibody solution consisting of a 1∶10,000 dilution of anti-DNP IgG antibody (Sigma) in Phosphate-buffered saline (PBS) containing 5.0% non-fat dry milk. The blots were then washed with PBS, 0.1% (v/v) Tween and incubated with the goat anti-rabbit IgG/HRP conjugate (1∶3,000 dilution in PBS/Milk) for 1 hour at room temperature. An enhanced chemiluminescence kit (Immobilon Western Chemiluminescent AP substrate, Millipore) was used for detection. To exclude unspecific reactions, in a control experiment, we omitted the incubation of IPG strips with DNPH. In this condition we did not detect any signal on PVDF membrane (data not shown).

### Image Acquisition and Analysis

For each muscle specimen (EDL and soleus from ten rats), 2D silver stained gels and 2D Western blots (2D-Oxyblot) were run in duplicate. Gel and Oxyblot images were digitized using the Epson expression 1680 PRO scanner and saved as TIFF files. Computer-aided 2D image analysis was carried out using ImageMaster 2D Platinum software version 6.0 (GE Healthcare) on both 2D silver staining gels (for differential expression analysis) and on 2D-Oxyblots (for differential carbonylation analysis). After automatic protein detection and matching, the gels were manually corrected to remove wrongly assigned or duplicated spots and image artifacts. Gel to gel variation was checked by a routine procedure that controls the coefficient of variation (CV). Average CV for all matched spots in our experimental series is less than 30%, indicating a typical variability according to Molloy et al [40]. The correlation coefficient of reproducibility of our gels was always higher than 0.89. Relative spot volume (%V = V single spot/V total spots, where V is the integration of the optical density over the spot area) was used during analysis in order to reduce experimental error. Spots detected in the 2D-Oxyblots and 2D silver stained gels were matched by computer-assisted image analysis using ImageMaster 2D Platinum 6.0 software. The immunoreactive spots whose %V variation *versus* control showed a significant *p*-value (*p*<0.05) and that exhibited at least a 1.8-fold decrease or increase in spot %V, were chosen for mass spectrometry (MS). Only the reproducible differences were taken into account. The %V of carbonylated spots on Oxyblots was normalized *versus* their respective spots visualized on silver stained gels.

### Protein Identification by Mass Spectrometry (MS)

Protein identification was carried out by peptide mass fingerprinting (PMF) on Ettan MALDI-TOF Pro mass spectometer (GE Healthcare) as previously described [41]. After visualization by colloidal Comassie staining protocol, spots were mechanically excised and destained with a solution of 2,5 mM ammonium bicarbonate in 50% ACN. The destained spots were subsequently dehydrated with ACN. A trypsin solution (0.25 µg/µl) in 50 mM ammonium bicarbonate was added for in-gel protein digestion which was carried out by overnight incubation at 37°C. Solutions containing digested peptides were recovered and 20 µl of 1% TFA 50% ACN were added to each spot and sonicated for 10 minutes to maximize peptide recovery. At the end, all recovered peptide solutions were combined and each spot was separately concentrated. Proteolytic peptides were mixed with CHCA matrix solution (5 mg/ml alpha-cyano-4-hydroxycinnamic acid (CHCA) in 0.1% TFA/70% ACN, v/v) in a 1∶1 ratio, and 2 µl of this mixture was spotted onto the MALDI target. Mass spectra were acquired automatically using the Ettan MALDI Evaluation software (GE Healthcare). Spectra were internally calibrated using the autoproteolysis peptides of trypsin (842.51 and 2211.10 Da). Protein identification by Peptide Mass Fingerprints search was performed using MASCOT version 2.2 as the search engine (Matrix Science, London, UK, http://www.matrixscience.com) through the Swiss-Prot/UniprotKB database. Taxonomy was limited to *Rattus norvegicus*, a mass tolerance of 100 ppm was allowed and the number of accepted missed cleavage sites was set to one. Alkylation of cysteine by carbamidomethylation was considered a fixed modification, while oxidation of methionine was considered as a possible modification. The criteria used to accept identifications included the extent of sequence coverage (at least 10%), the number of matched peptides (at least 5) and a probabilistic score at p<0.05. Protein scores greater than 69 are significant (*p*<0.05).

### Western Blot Analysis of Proteomic Candidates

For 1D-GE, 20 µg of protein extracts were separated by 12% SDS-PAGE and transferred onto a PVDF membrane (Millipore). The relative amount of fructose-bisphosphate aldolase, enolase and actin proteins were assessed by Western blot with appropriate antibodies (Santa Cruz Biotechnology). The blots were subjected to densitometric analysis by using Quantity One Software (Bio-Rad). Subsequently these blots were stained with Coomassie brilliant blue R-250. For the quantification, the intensity of the immunostained bands was normalized with the total protein intensities measured by Coomassie brilliant blue R-250 from the same blot. Statistical analysis of the data was performed by Student's t-test; p-values <0.05 were considered statistically significant.

### TBARS Measurement

As a lipid peroxidation marker, we measured thiobarbituric acid reactive substances (TBARS) ((Cayman Chemical Company, Ann Arbor, MI, USA). Malondialdehyde (MDA), was expressed in nanomoles per mg of proteins.

### Statistical Analysis

All data are presented as Mean±Standard deviation. Differences between C and ASE rats were determined using Student’s two tailed independent t-test. *P*-values ≤0.05% were considered significant.

## Results

### Effects of an Acute Swimming Exercise on Muscle Protein Expression

**Figure 1:**
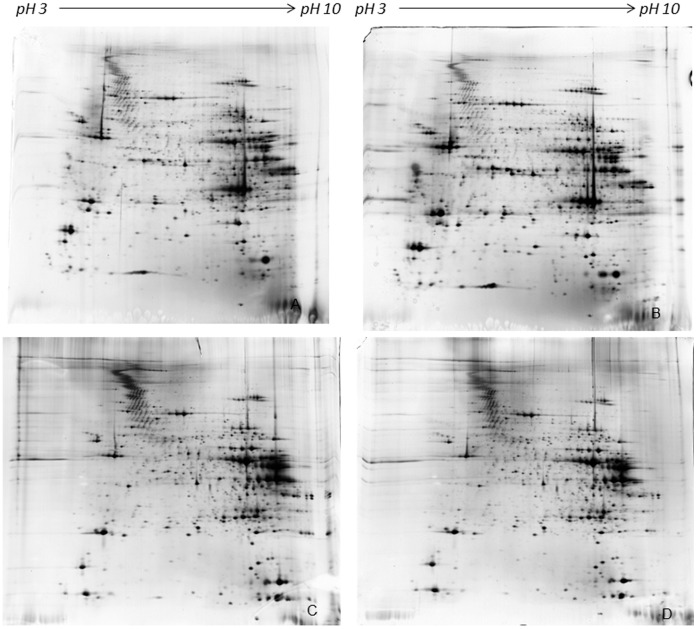
Representative silver stained gels of Soleus (control in panel A, ASE in panel B) and EDL (control in panel C, ASE in panel D) muscles. Muscle proteins were separated by non-linear pH 3–10 gradient and 9–16% polyacrylamide linear gradient. Computer-aided 2D image analysis, carried out using ImageMaster 2D Platinum software version 6.0, does not show any significant difference between control and ASE muscles protein expression.

Soleus and EDL proteins, purified from control and ASE groups, were compared by 2D-GE analysis. The proteins were separated and resolved in all areas of the gels and an average of 1650 and 1720 spots were detected in Soleus and EDL muscles respectively using ImageMaster 2D Platinum software version 6.0. The comparison of control and ASE groups, showed the absence of significant (p≤0.05) differences in relative spot volumes, thus indicating that, in our experimental conditions, a single bout of acute swimming does not affect protein expression in either muscles (Fig. 1).

### ASE-induced Protein Carbonylation Modification

**Figure 2:**
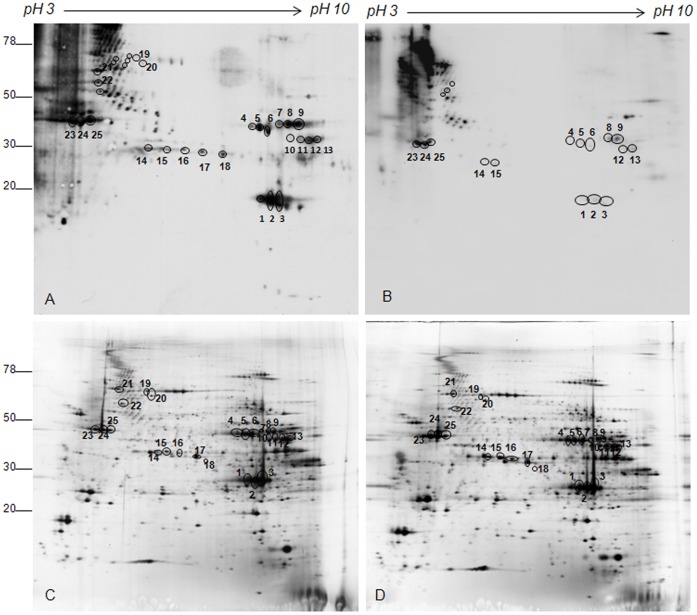
Representative Oxyblot and silver stained gels of Soleus muscle from control and ASE animals. Muscle proteins from control (A) and ASE animals (B) were separated by IEF and carbonylated proteins were derivatized with DNPH in the strip (18 cm, 3–10 NL). Second dimension was performed in 9–16% polyacrylamide linear gradient. In panel C and D the corresponding silver stained gels are shown. Circles indicate spots where carbonylation was affected by ASE. Circles and numbers indicate spots identified by MS.

To find carbonylated proteins arising as a consequence of ASE, we performed an in-strip derivatization with DNPH followed by SDS-PAGE and Oxyblot using the procedure previously described [29].Only the reproducible differences were taken into account. Fig. 2 shows representative images of Soleus muscle Oxyblots obtained from control (A) and ASE animals (B). The analysis found 31 carbonyl-modified spots in Soleus muscle from sedentary rats, 13 of which were not detectable in Oxyblots obtained from Soleus muscle of ASE subjects. Since the computer analysis of the silver stained gels did not reveal any difference in corresponding protein expression level between control and ASE muscles (Fig. 2 panels C and D), the decreased carbonylation level indicates that acute swimming exercise induces a reduction in carbonyl content of target proteins.

**Figure 3:**
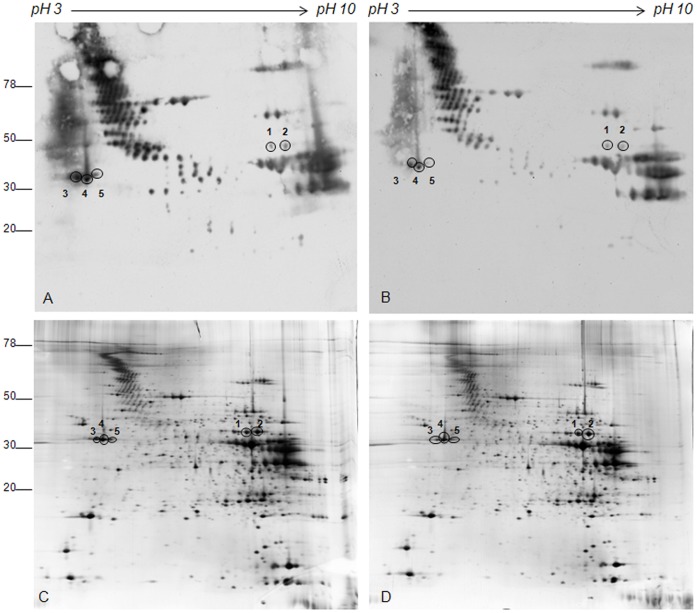
Representative Oxyblot and silver stained gels of EDL muscle from control and ASE animals. Muscle proteins from control (A) and ASE animals (B) were separated by IEF and carbonylated proteins were derivatized with DNPH in the strip (18 cm, 3–10 NL). Second dimension was performed in 9–16% polyacrylamide linear gradient. In panel C and D the corresponding silver stained gels are shown. Circles indicate spots where carbonylation was affected by ASE. Circles and numbers indicate spots identified by MS.

In EDL muscle, acute swimming induced a variation in carbonyl content of only five proteins (Fig. 3, panels A and B). As in Soleus muscles, these variations are characterized by a decrease of the carbonylation level in ASE compared to controls, while no change in the expression level of corresponding spots was observed (Fig. 3, panels C and D).

### MS Spectrometry Identification of Carbonylated Proteins

To identify the 36 spots that show a variation in carbonylation level (31 spots from Soleus and 5 spots from EDL), preparative gels were performed and colloidal Coomassie-stained. The 2D-silver stained images were then matched to the images of preparative gels in order to excide the selected spots for mass spectrometry analysis. Due to imprecise overlapping between the two types of images, six carbonylated spots, clearly visible in the gel acidic regions of Soleus 2D-Oxyblot and in the corresponding silver stained gels, could not be localized with certainty and were left out, therefore 25 and 5 protein spots were identified in Soleus and EDL muscle, respectively. These spots are indicated by circles and numbers in Fig. 2 and Fig. 3 (panel C and D) and in Figures S1 and S2. Table 1 and Table S1 of Supporting information reports protein identities and MS data.

**Table 1:** Identity of Soleus and EDL muscle proteins whose carbonylation is affected by acute swimming.

Spot N°^a^	Accession number^b^	Protein name	Fold change^c^(%V control/%V ASE)
**Soleus**			
1	P14141	Carbonic anhydrase 3	12
2	P14141	Carbonic anhydrase 3	14
3	P14141	Carbonic anhydrase 3	11
4	P00564	Creatine kinase M-type	3
5	P00564	Creatine kinase M-type	5
6	P00564	Creatine kinase M-type	11
7	P09605	Creatine kinase S-type, mitochondrial	Only in control
8	P09605	Creatine kinase S-type, mitochondrial	1.7
9	P09605	Creatine kinase S-type, mitochondrial	1.8
10	P05065	Fructose-bisphosphate aldolase A	Only in control
11	P05065	Fructose-bisphosphate aldolase A	Only in control
12	P05065	Fructose-bisphosphate aldolase A	3.4
13	P05065	Fructose-bisphosphate aldolase A	2.4
14	Q7TNB2	Troponin T, slow skeletal muscle	14
15	Q7TNB2	Troponin T, slow skeletal muscle	4
16	Q7TNB2	Troponin T, slow skeletal muscle	Only in control
17	Q7TNB2	Troponin T, slow skeletal muscle	Only in control
18	O88989	Malate dehydrogenase, cytoplasmic	Only in control
19	Q07439	Heat shock 70 kDa protein 1A/1B	Only in control
20	P08461	Dihydrolipoyllysine-residue acetyltransferase componentof pyruvate dehydrogenase complex, mitochondrial	Only in control
21	P63018	Heat shock cognate 71 kDa protein	Only in control
22	P63018	Heat shock cognate 71 kDa protein	Only in control
23	P68136	Actin, alpha skeletal muscle	2
24	P68136	Actin, alpha skeletal muscle	1.8
25	P68136	Actin, alpha skeletal muscle	1.8
**EDL**			
1	P15429	Beta-enolase	1.8
2	P15429	Beta-enolase	2.2
3	P68136	Actin, alpha skeletal muscle	2.1
4	P68136	Actin, alpha skeletal muscle	3
5	P68136	Actin, alpha skeletal muscle	1.9

a Spot numbers match those reported in the representative 2-DE images shown in Fig. 2 (Soleus muscle) and in Fig. 3 (EDL muscle).

b Accession number in UniProtKB/Swiss-Prot.

c Fold change control *vs.* ASE was calculated as follow: %V_control_/%V_ASE_. (*p*<0.05).

Noteworthy, among the proteins displaying a reduced or absent carbonyl content following ASE, two proteins were functionally related to the contractile apparatus: Troponin T, involved in the regulation of muscle contraction, and alpha-Actin, a major constituent of the contractile apparatus. Troponin T carbonylation level was decreased in ASE-Soleus muscles (spots 14–17), whereas alpha-Actin in both Soleus (spots 23–25) and EDL (spots 3–5) following ASE. In Soleus, also the cytoplasmic Carbonic Anhydrase (spots 1–3), which is expressed at high levels in oxidative slow-twitch skeletal muscle [42], showed a reduced level of carbonylation after acute swimming exercise. Moreover, the carbonyl-content of some metabolic enzymes was decreased or undetectable in ASE animals: Creatine Kinase M-type (spots 4–6), mitochondrial Creatine Kinase S-type (spots 7–9), Fructose-bisphosphate Aldolase A (spots 10–13) and cytoplasmic Malate Dehydrogenase (spot 18) in Soleus and two isoforms of beta-Enolase (spots 1, 2) in EDL.

### Western Blot Analyses of Representative Proteins

**Figure 4:**
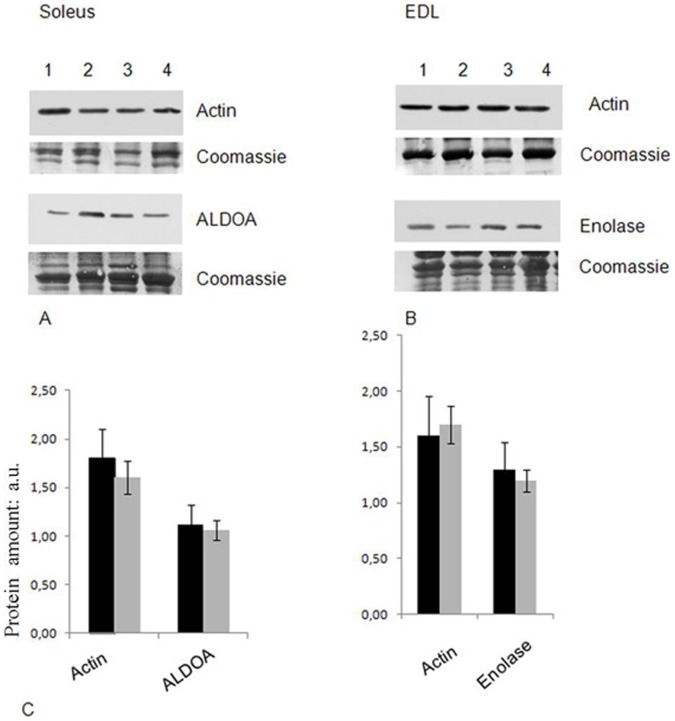
Validation of proteomic results. Protein amount of representative proteins in Soleus (panel A: actin and fructose-bisphosphate Aldolase A ) and EDL (Panel B: Actin and Enolase) was checked by WB with corresponding antibodies. Intensity of immunostained bands was normalized with the total protein intensities measured from the same blot stained with Coomassie brilliant blue (a representative band of the lane is reported); lanes 1, 2 and 3, 4 represent control and ASE animals, respectively. In panel C, the mean value of expression variation (arbitrary units) is reported (black bars: control animals; grey bars: ASE animals). Two-tailed, non-paired Student’s t-test was was performed. * indicates a p-value <0.05.

As described above, no difference in expression level of the identified proteins was observed in silver stained gels obtained from control and ASE animals. To confirm that the reduced carbonylation level of the identified proteins was independent on a reduced protein expression, we evaluated the level of specific proteins by western blot analysis. In particular, we analyzed the expression level of three carbonylated proteins whose carbonylation decreased after ASE: Fructose-bisphosphate aldolase A (spots 10–13 in Soleus muscle), alpha-Actin (spots 23–25 in Soleus and 3–4 in EDL) and beta-Enolase (spots 1–2 in EDL). The results reported in Fig. 4 show that the expression of these proteins is unaffected by ASE, thus confirming a decrease in their carbonylation amount.

### Lipid Peroxidation

**Figure 5:**
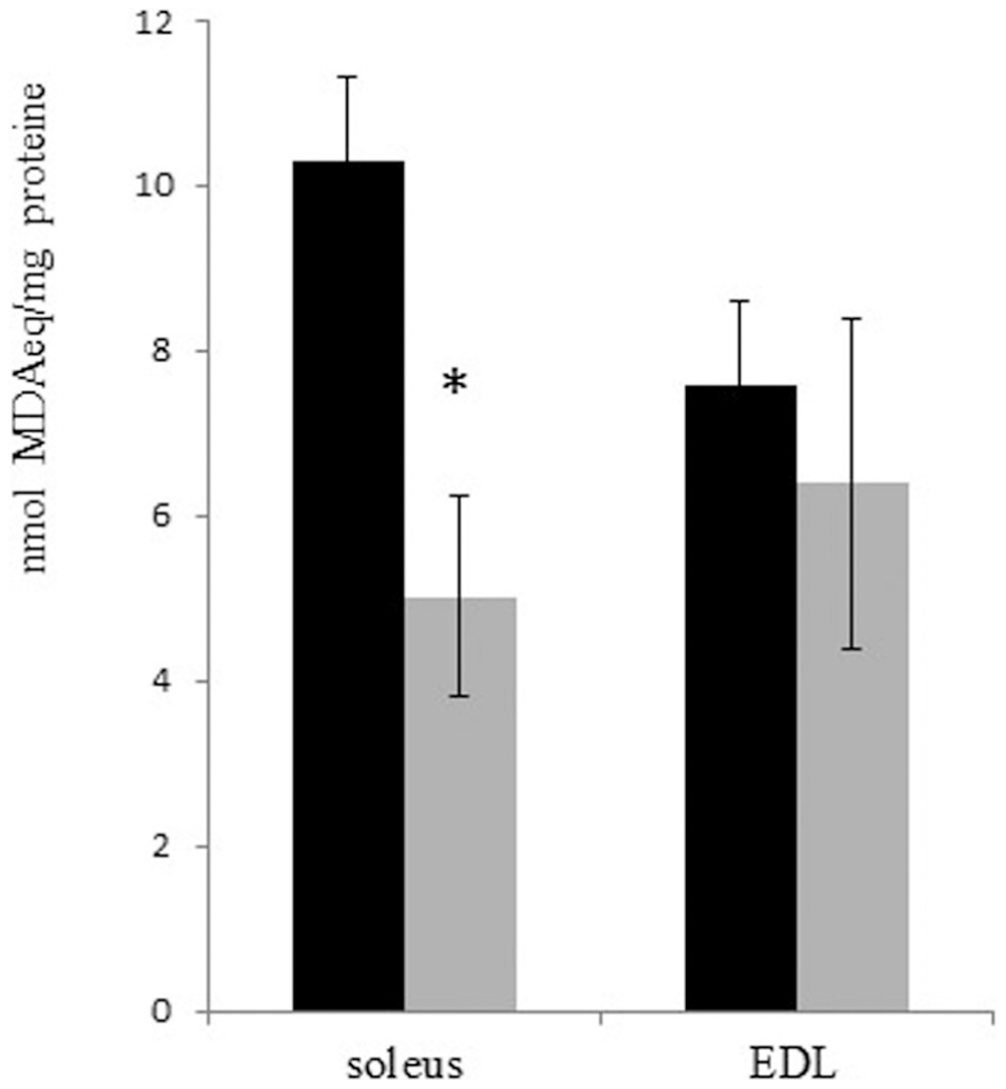
Lipid peroxidation in Soleus and EDL muscles. Lipid peroxidation was evaluated by measuring the malondialdehyde levels. Black bars: control animals; gray bars: ASE animals. Two-tailed, non- paired Student’s t-test was performed. * indicates a p-value <0.05.

Lipid peroxidation was evaluated in order to assess an oxidative stress marker independent from protein carbonylation. As reported in Fig. 5, the level of malondialdehyde (a lipid peroxidation product) was significantly reduced by approximately 50% in Soleus muscle following ASE, whereas no significant difference was observed in EDL muscle.

## Discussion

Although proteomic approaches were used in several studies to verify muscle protein expression variation induced by exercise, a complete and comprehensive overview of this field is not currently available, due to the great variability in animal models, training programs and muscle types considered. In a previous paper, we found that a 10-weeks aerobic moderate exercise training affected the global protein expression in Tibials Anterior muscle, which is mostly composed of fast-twitch fibers. We found that many glycolytic enzymes, such as alpha-Enolase, beta-Enolase and Triosephosphate isomerase, were down-regulated, as a result of the partial switch to aerobic metabolism [23]. Using a similar approach, Burniston [43] identified 11 proteins affected by training in Plantaris muscle. Among these, alpha-Enolase showed the same trend of our study. In addition, Guelfi *et al*. [44] investigated protein expression profile in Gastrocnemius muscle after a single bout of weighted swimming, a high-intensity physical activity. They found that only four proteins showed variation in expression level. The effect of a single bout of incremental exercise was investigated by Gandra *et al* [45] who found that four proteins showed a variation in expression level after three hours of recovery, but only two maintained such differential expression following 24 hours of recovery. Taken together, these data suggest that single bouts of exercise, as opposed to sustained training, involve few alterations of protein expression, which may reflect transient – as opposed to stable – modifications. In our experimental conditions, we found no variation in protein expression level in Soleus and EDL in ASE animals. Using the same exercise protocol, we previously showed that de novo protein synthesis occurred in liver [35]. However, the most striking finding is the decrease in the carbonylation level of muscle proteins following ASE. Such decrease was more pronounced in Soleus than in EDL. Moreover lipid peroxidation was also found to be reduced in Soleus muscle. In a previous paper, we demonstrated that the basal level of protein carbonylation is higher in Soleus than in Tibialis Anterior and that this basal level is moderately increased after a controlled training program in both muscles [23]. Since Soleus has an oxidative metabolism, it may produce, even in basal conditions, a higher amount of RONS and have a higher level of built-in antioxidant protection. This is supported by the fact that anti-oxidant enzymes and the inducible form of HSP70 protein were expressed to a higher extent in Soleus than in Tibialis Anterior in control rats [46]. Thus, the marked decrease in carbonylated protein levels observed in Soleus following ASE could reflect the increased ability of Soleus to cope with the sudden increase in RONS, but may also imply other still unexplored defense mechanisms towards oxidative stress. In fact, a single bout of acute exercise in untrained animals is more stressing than a controlled training, hence it can activate novel mechanisms involved in stress defense, protein turnover or signaling. The latter was investigated in a study that deals with S-nitrosylation, a different type of protein oxidation, where it was demonstrated that a single bout of exercise reverses insulin sensitivity in diet-induced obese rats by improving the insulin signaling pathway [47]. Concerning the decrease in carbonylated protein levels observed in our experimental model, four hypotheses may be advanced: (i) the most conservative hypothesis suggests that the acute bout of exercise greatly up-regulates antioxidant defenses, resulting not only in the ability to keep in check the sudden increase of RONS, but also in reducing pre-existing oxidative modifications. This hypothesis accounts also for the observed reduction in lipid peroxidation in ASE Soleus; however, it should be stressed that long-term training results in the opposite outcome (1); (ii) the most intriguing hypothesis, which counteracts the deep-rooted idea that carbonylation is an irreversible modification, is in keeping with Wong *et al.* [19], who propose that protein carbonylation and decarbonylation serve as a mechanism of signal transduction and suggest a thiol-dependent reduction mechanism for the decarbonylation system; (iii) another hypothesis is based on the possibility that acute exercise promotes protein turnover; in fact oxidized proteins can be degraded directly by the 20S proteasome without ubiquitination or rapidly degraded by proteases [19]; (iv) finally, the decrease in protein carbonylation might reflect a further oxidation step whereby carbonylated aldehyde or ketone are oxidized to carboxylic acid, which does not react with DNPH [48]. It should be stressed, however, that this last hypothesis does not account for the decrease in lipid peroxidation in Soleus muscle from ASE rats and that further investigations are needed in order to explore, among other things, the very meaning of protein carbonylation. It is paramount that the present results and the coexistence of at least four possible interpretations, that may account for the decrease in protein carbonylation following ASE, challenge the idea that proteins may just constitute a sink for otherwise damaging oxidative modifications, as well as the idea that short bursts of exercise in untrained animals increase RONS formation. In fact, it should be reminded that myosin heavy chain binding to actin requires that the two proteins are in a reduced state, since the oxidation of one of the two proteins causes the disassembly of the actomyosin complex leading to reduced contraction [49], [50]. Hence, basal carbonylation levels of contractile proteins may contribute to a partial and reversible down-regulation of the contractile apparatus during steady-state conditions; the same may be true for some metabolic enzymes involved in energy usage.

Taken together, these findings strongly support the idea that the effects of acute exercise differ from those induced by a long-lasting training protocol where increased carbonylation level has been shown [23], [43]. Moreover, our results suggest the existence of still unknown and unexpected defence mechanisms leading to cycles of carbonylated/decarbonylated protein turnover when a single bout of acute exercise is applied.

## Supporting Information

Figure S1 Colloidal Coomassie stained gel from a pool of control and ASE of soleus muscle proteins. Annotations indicated spots that correspond to carbonylated proteins. These spots were picked for MS.
(TIF)

Figure S2 Colloidal Coomassie stained gel of a pool of control and ASE of EDL muscle proteins. Annotations indicated spots that correspond to carbonylated proteins. These spots were picked for MS.
(TIF)

Table S1 MALDI-TOF analysis of muscle protein spots which carbonylation is affected by ASE.
(DOCX)
