# Supplementary figures and images for: Proteomic and Carbonylation Profile Analysis of Rat Skeletal Muscles following Acute Swimming Exercise
Source: PLoS One. 2013 Aug 13;8(8):e71839. doi: 10.1371/journal.pone.0071839 (PMC3742498; doi:10.1371/journal.pone.0071839)

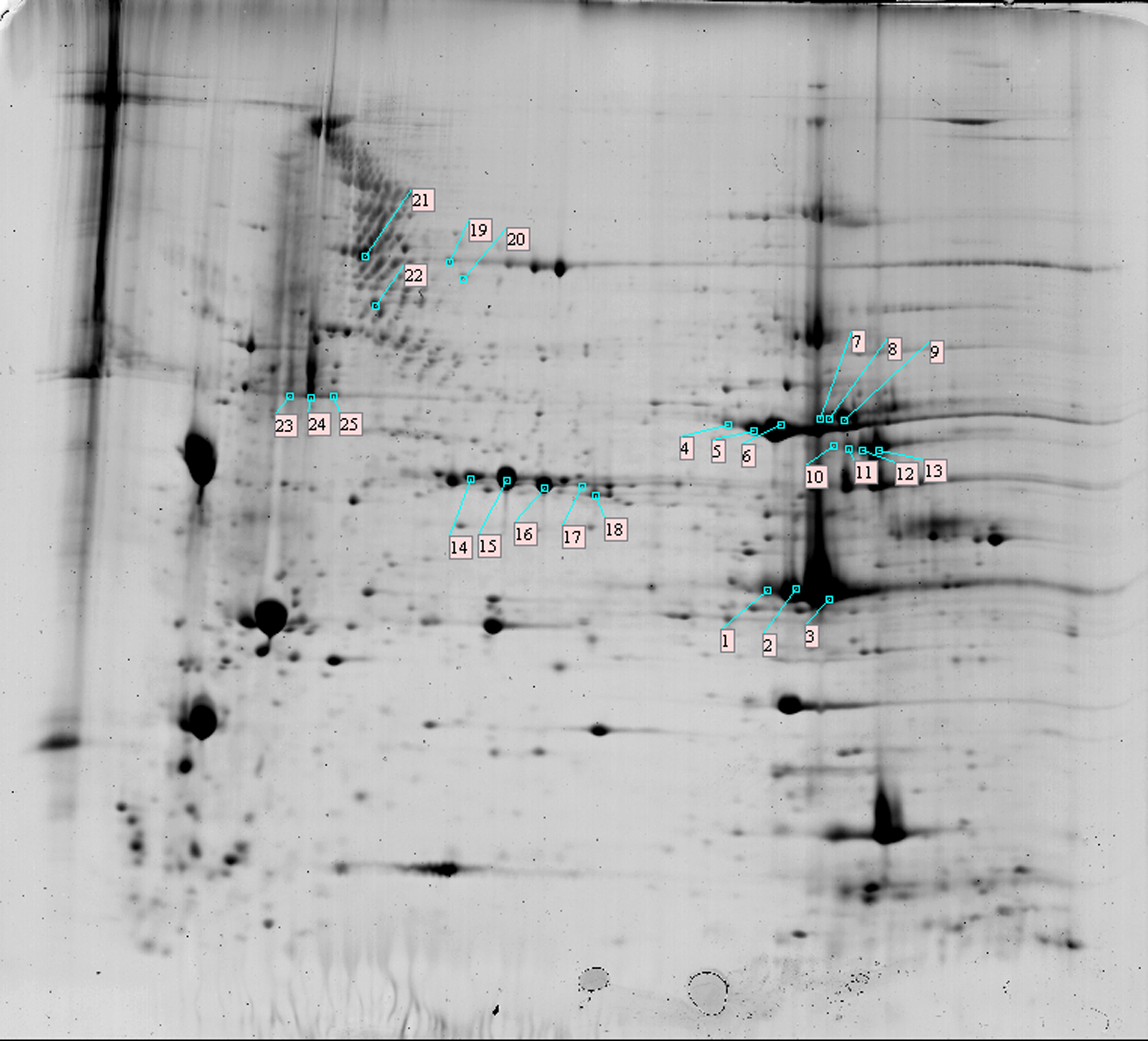

Supplement: Figure S1 — Colloidal Coomassie stained gel from a pool of control and ASE of soleus muscle proteins. Annotations indicated spots that correspond to carbonylated proteins. These spots were picked for MS. (TIF) [file pone.0071839.s001.tif]

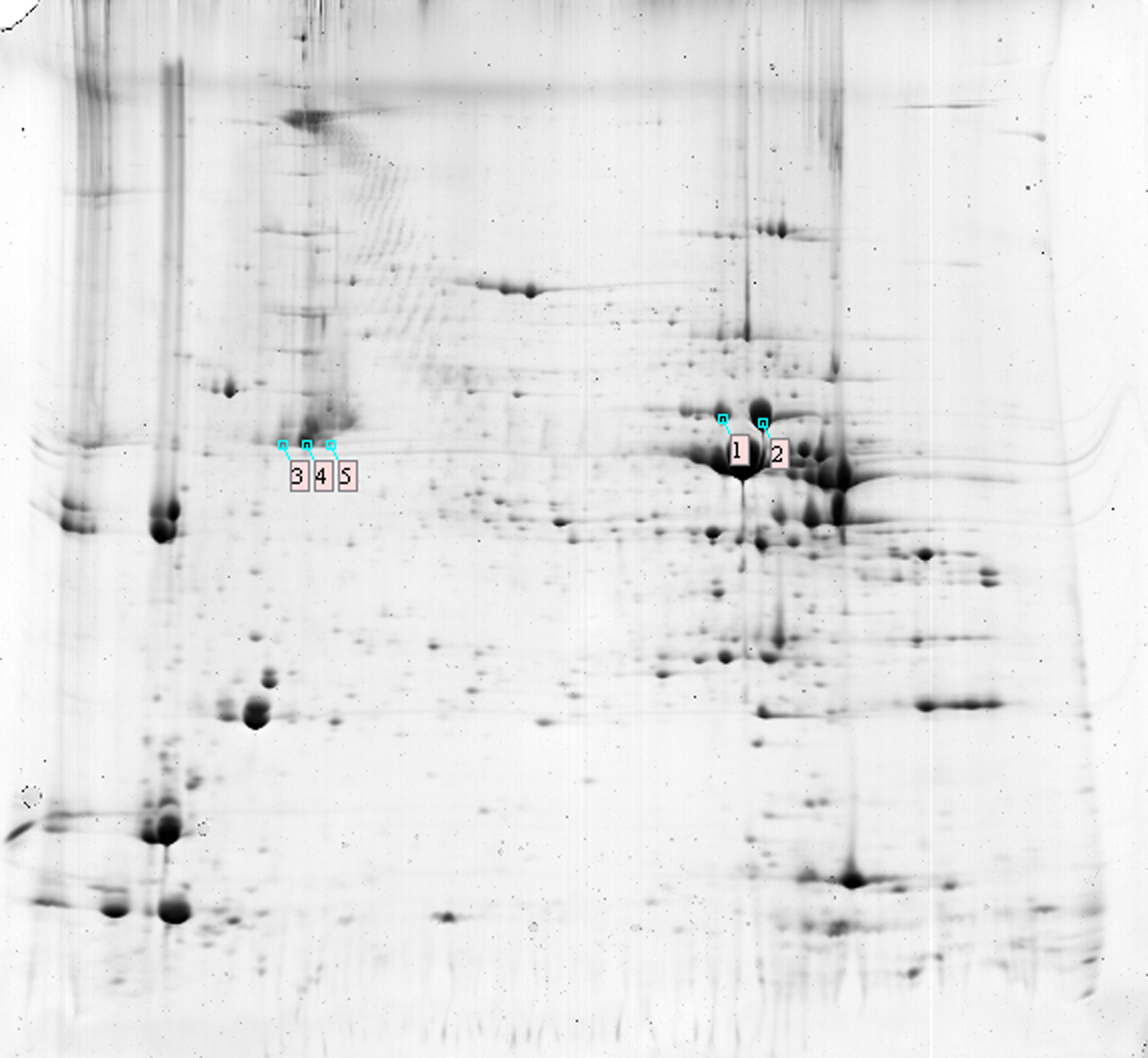

Supplement: Figure S2 — Colloidal Coomassie stained gel of a pool of control and ASE of EDL muscle proteins. Annotations indicated spots that correspond to carbonylated proteins. These spots were picked for MS. (TIF) [file pone.0071839.s002.tif]
