# Supplementary material for: Proteomic and Carbonylation Profile Analysis of Rat Skeletal Muscles following Acute Swimming Exercise
Source: PLoS One. 2013 Aug 13;8(8):e71839. doi: 10.1371/journal.pone.0071839 (PMC3742498; doi:10.1371/journal.pone.0071839)
Supplement: Table S1 — MALDI-TOF analysis of muscle protein spots which carbonylation is affected by ASE. (DOCX) [file pone.0071839.s003.docx]

**Table S1**. MALDI-TOF analysis of muscle protein spots which carbonylation is affected by ASE

| **Spot N°^a^** | **Accession number^b^** | **Protein name** | **MW(Da)/pI** | **Matched peptides/**  **searched peptides^c^** | **Sequence coverage^d^** | **Score^e^** |
| --- | --- | --- | --- | --- | --- | --- |
| **Soleus** | | | |  |  |  |
| 1 | **P14141** | Carbonic anhydrase 3 | 29698/ 6.89 | 7/9 | 29% | 116 |
| 2 | **P14141** | Carbonic anhydrase 3 | 29698/ 6.89 | 9/13 | 39% | 114 |
| 3 | **P14141** | Carbonic anhydrase 3 | 29698/ 6.89 | 14/23 | 70% | 217 |
| 4 | P00564 | **Creatine kinase M-type** | 43246/ 6.58 | 8/12 | 24% | 107 |
| 5 | P00564 | **Creatine kinase M-type** | 43246/ 6.58 | 8/11 | 24% | 117 |
| 6 | P00564 | **Creatine kinase M-type** | 43246/ 6.58 | 9/14 | 31% | 121 |
| 7 | P09605 | **Creatine kinase S-type, mitochondrial** | 47811/8.76 | 16/42 | 37% | 126 |
| 8 | P09605 | **Creatine kinase S-type, mitochondrial** | 47811/8.76 | 13/29 | 26% | 107 |
| 9 | P09605 | **Creatine kinase S-type, mitochondrial** | 47811/8.76 | 16/28 | 40% | 159 |
| 10 | P05065 | Fructose-bisphosphate aldolase A | 39783/ 8.31 | 8//10 | 28% | 127 |
| 11 | P05065 | Fructose-bisphosphate aldolase A | 39783/ 8.31 | 11/17 | 28 % | 153 |
| 12 | P05065 | Fructose-bisphosphate aldolase A | 39783/ 8.31 | 13/21 | 40% | 167 |
| 13 | P05065 | Fructose-bisphosphate aldolase A | 39783/ 8.31 | 8/14 | 35% | 110 |
| 14 | Q7TNB2 | **Troponin T, slow skeletal muscle** | 31196/5.85 | 8/9 | 37% | 115 |
| 15 | Q7TNB2 | **Troponin T, slow skeletal muscle** | 31196/5.85 | 12/22 | 42% | 118 |
| 16 | Q7TNB2 | **Troponin T, slow skeletal muscle** | 31196/5.85 | 10/17 | 39% | 105 |
| 17 | Q7TNB2 | **Troponin T, slow skeletal muscle** | 31196/5.85 | 9/13 | 34% | 114 |
| 18 | O88989 | **Malate dehydrogenase, cytoplasmic** | 36631/ 6.16 | 8/13 | 29% | 104 |
| 19 | Q07439 | **Heat shock 70 kDa protein 1A/1B** | 70427/ 5.61 | 11/14 | 24% | 143 |
| 20 | P08461 | Dihydrolipoyllysine-residue acetyltransferase component of pyruvate dehydrogenase complex, mitochondrial | 67637/ 8.76 | 8/9 | 13% | 82 |
| 21 | **P63018** | Heat shock cognate 71 kDa protein | 71055/5.37 | 14/19 | 31% | 169 |
| 22 | **P63018** | **Heat shock cognate 71 kDa protein** | 61088/ 5.9 | 9/16 | 24% | 100 |
| 23 | **P68136** | Actin, alpha skeletal muscle | 42366/5.23 | 6/8 | 20% | 89 |
| 24 | **P68136** | Actin, alpha skeletal muscle | 442366/5.23 | 7/10 | 23% | 100 |
| 25 | **P68136** | Actin, alpha skeletal muscle | 42366/5.23 | 7/10 | 23% | 100 |
| **EDL** | | | |  |  |  |
| 1 | **P15429** | Beta-enolase | 47326/7.08 | 10/14 | 29% | 114 |
| 2 | **P15429** | Beta-enolase | 47326/ 7.08 | 15/23 | 34% | 130 |
| 3 | **P68136** | Actin, alpha skeletal muscle | 42366/5.23 | 6/8 | 20% | 89 |
| 4 | **P68136** | Actin, alpha skeletal muscle | 442366/5.23 | 8/10 | 24% | 107 |
| 5 | **P68136** | Actin, alpha skeletal muscle | 42366/5.23 | 7/10 | 26% | 120 |

^a^ Spot numbers match those reported in the representative 2D-GE images shown in Fig. 2 and 3 (panel C and D)

^b^ Accession number in UniProtKB/Swiss-Prot.

^c^ Number of matched peptides correspond to peptide masses matching the top hit from Ms-Fit PMF;

^d^ Sequence coverage indicates [number of the identified residues/total number of amino acid residues in the protein sequence] × 100%.

^e^ Score corresponds to MASCOT score (MatrixScience, London, UK; http.//www.matrixscience.com). Protein scores greater than 69 are significant (*p*<0.05).
